# Supplementary material for: Newly discovered Synechococcus sp. PCC 11901 is a robust cyanobacterial strain for high biomass production
Source: Commun Biol. 2020 May 7;3:215. doi: 10.1038/s42003-020-0910-8 (PMC7205611; doi:10.1038/s42003-020-0910-8)
Supplement: Supplementary file 1 — Supplementary Information [file 42003_2020_910_MOESM1_ESM.pdf]

Supplementary Figures

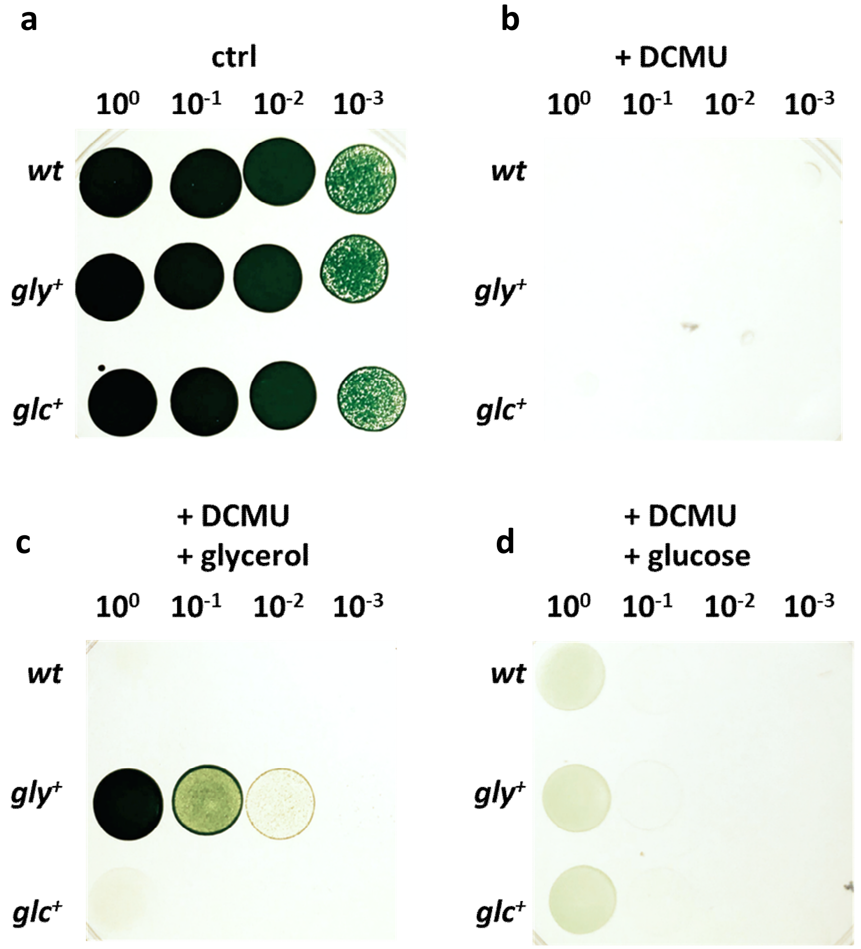

Supplementary Figure 1. Glycerol and glucose tolerance of PCC 11901 and photoheterotrophy analysis. Wild-type *(wt)*, glycerol *(gly$^+$)* and glucose *(glc$^+$)* adapted strains were grown to OD_{730} = 5 and subsequently diluted. Dilutions were transferred onto solid AD7 medium without any additives **(a)**, with 10 µM of herbicide DCMU **(b)**, 10 µM DCMU and 10 mM glycerol **(c)** and finally 10 µM DCMU and 0.15 % glucose **(d)**.

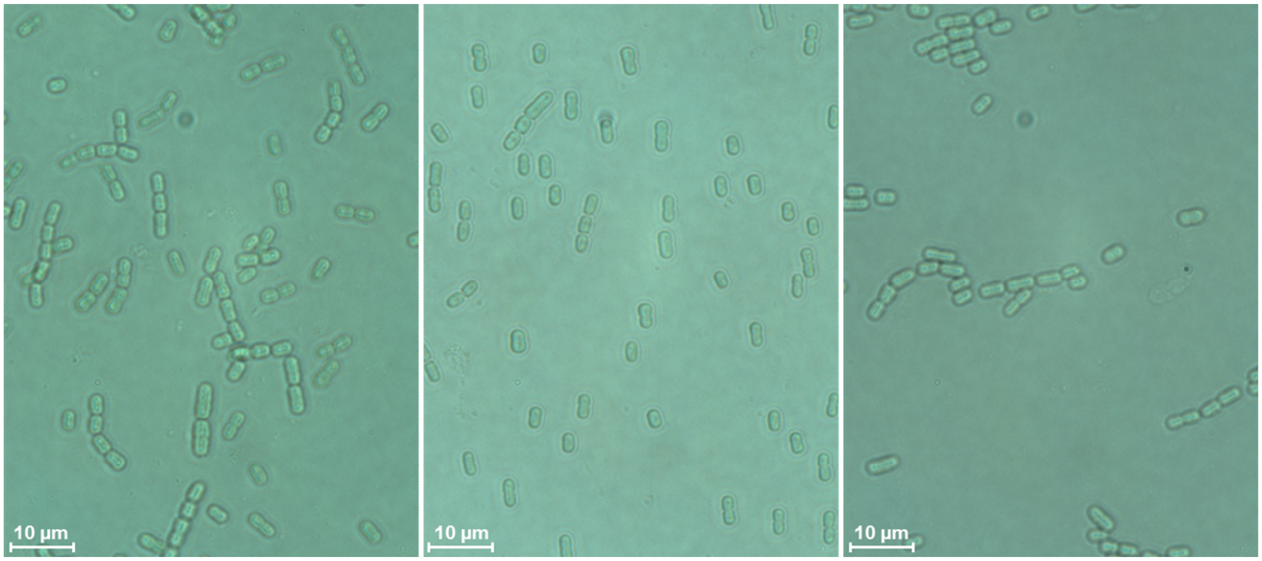

Supplementary Figure 2. Bright-field microscopy images of the PCC 11901 strain (magnification 1000x). Samples were collected at OD_{730} ≈5

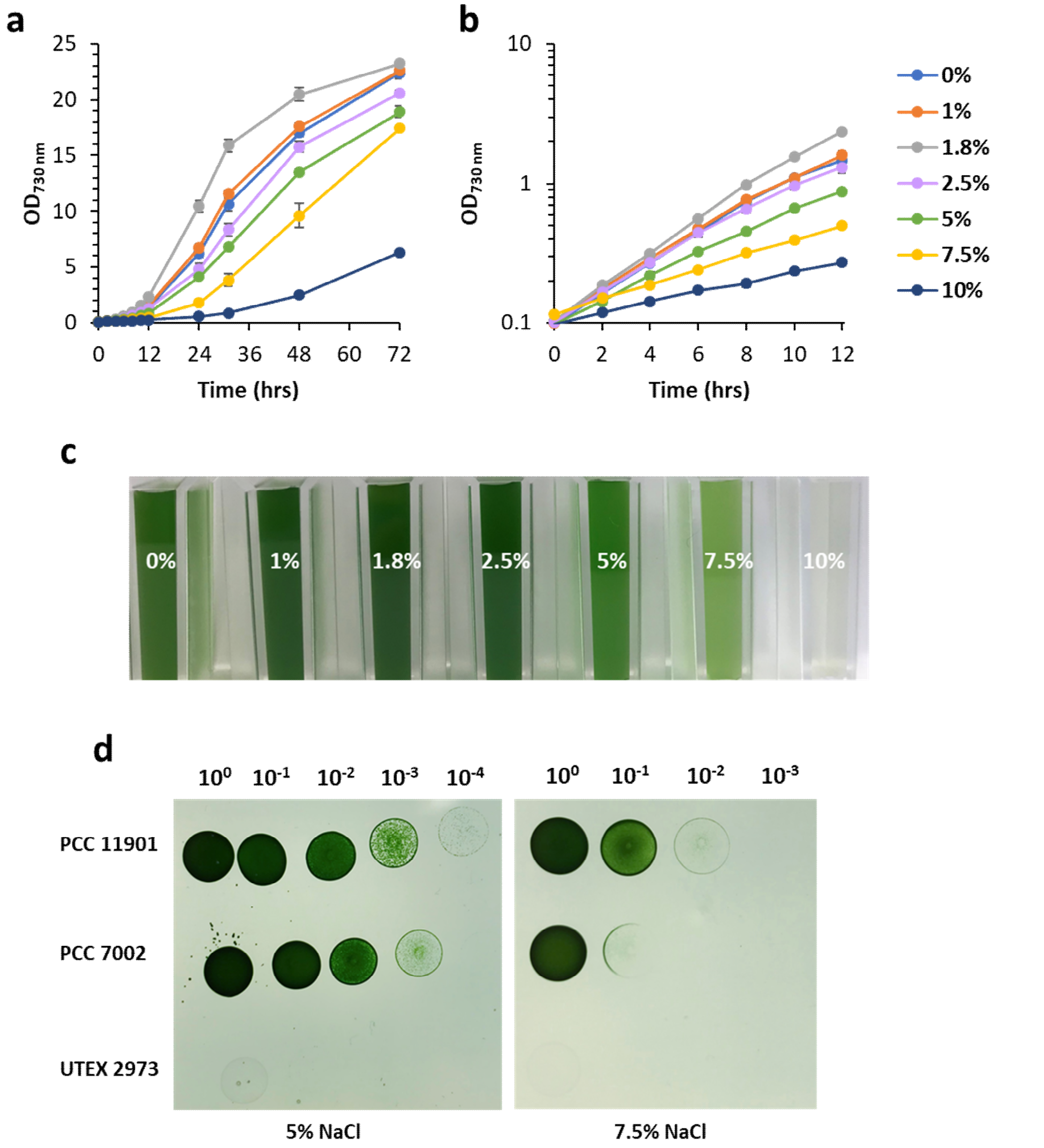

Supplementary Figure 3. Salt tolerance analysis of PCC 11901 strain. **(a)** Cultures were grown in triplicates for 72 hours at 38 °C, 1% CO_2, 225 rpm shaking, constant illumination of 300 µmol photons·m^{-2}·s^{-1} using AD7 media with varying sodium chloride (w/v) concentrations. **(b)** In the exponential growth phase, measurements were taken in 2-hours intervals. Time points within exponential phase were used for calculating the average growth rate. Average OD_{730} was calculated as mean of n=3 biological replicates ± standard deviation. **(c)** Culture samples display collected after 24 hours of cultivation. **(d)** Salt tolerance of PCC 11901, PCC 7002 and UTEX 2973 strains. Cultures grown in regular media were grown to OD=5, subsequently diluted and plated on solid AD7 medium with 5% and 7.5% sodium chloride. Plates were incubated at 100 µmol photons·m^{-2}·s^{-1} and 38 °C for 7 days.

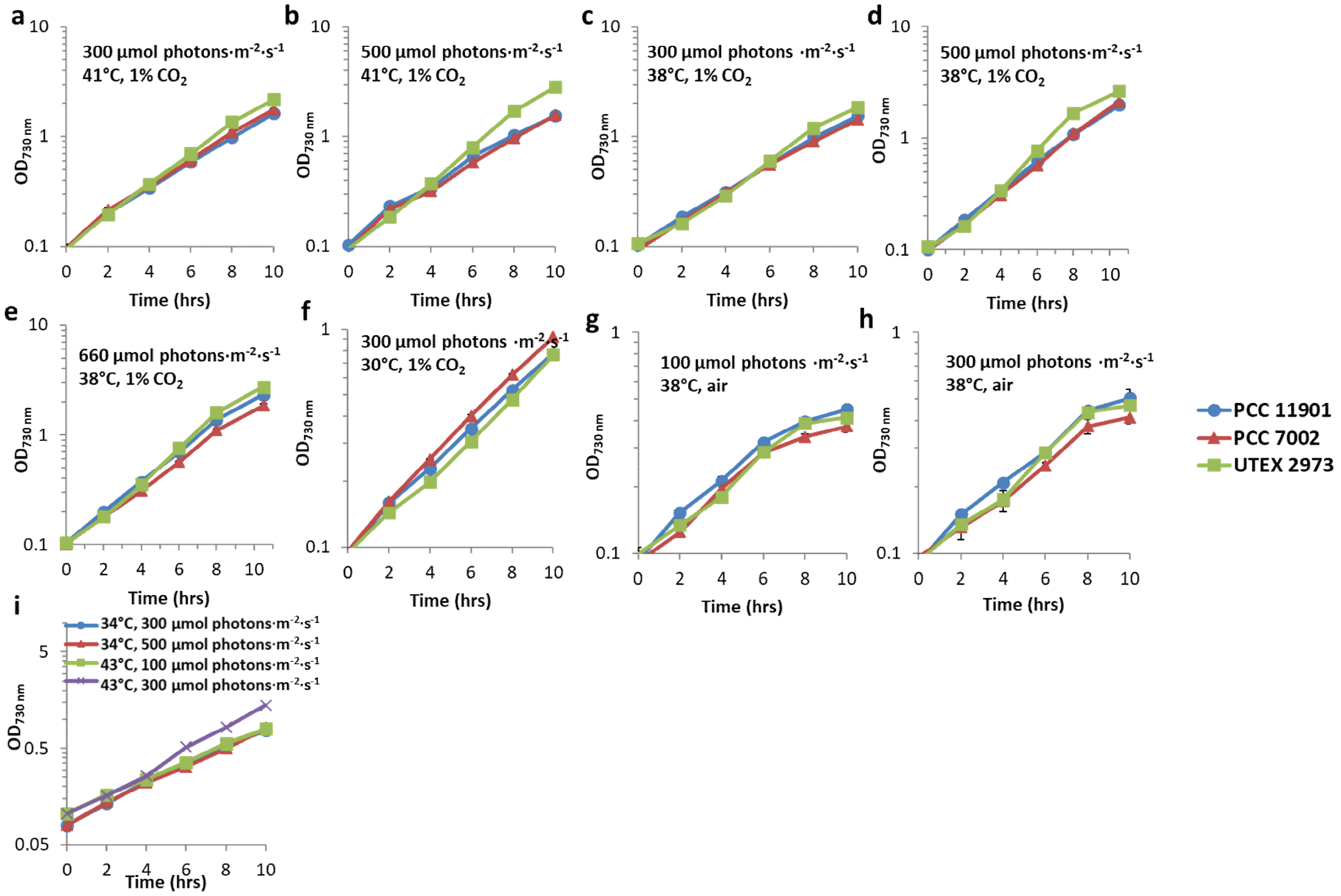

Supplementary Figure 4. Growth performance comparison of PCC 11901, PCC 7002 and UTEX 2973 strains in different conditions. Strains, depending on requirements were grown in either AD7 or BG-11 medium, under constant illumination using LED RGB light 4:2:1 ratio setting **(a-d, f-h)**, except for growth curve **(e)** where red and blue LEDs only were used at 1:1 ratio. All strains were inoculated to initial OD_{730} of approximately 0.1. Cultures were grown in triplicates and OD_{730} measurements were taken in 2-hours intervals. Time-points within exponential growth phase only were used for calculating average doubling time. In blue growth curves of PCC 11901, in red PCC 7002 and in green UTEX 2973. Average OD_{730} was calculated as mean of n=3 biological replicates ± standard deviation. **(i)** Growth performance of PCC 11901 strain in additional light and temperature conditions.

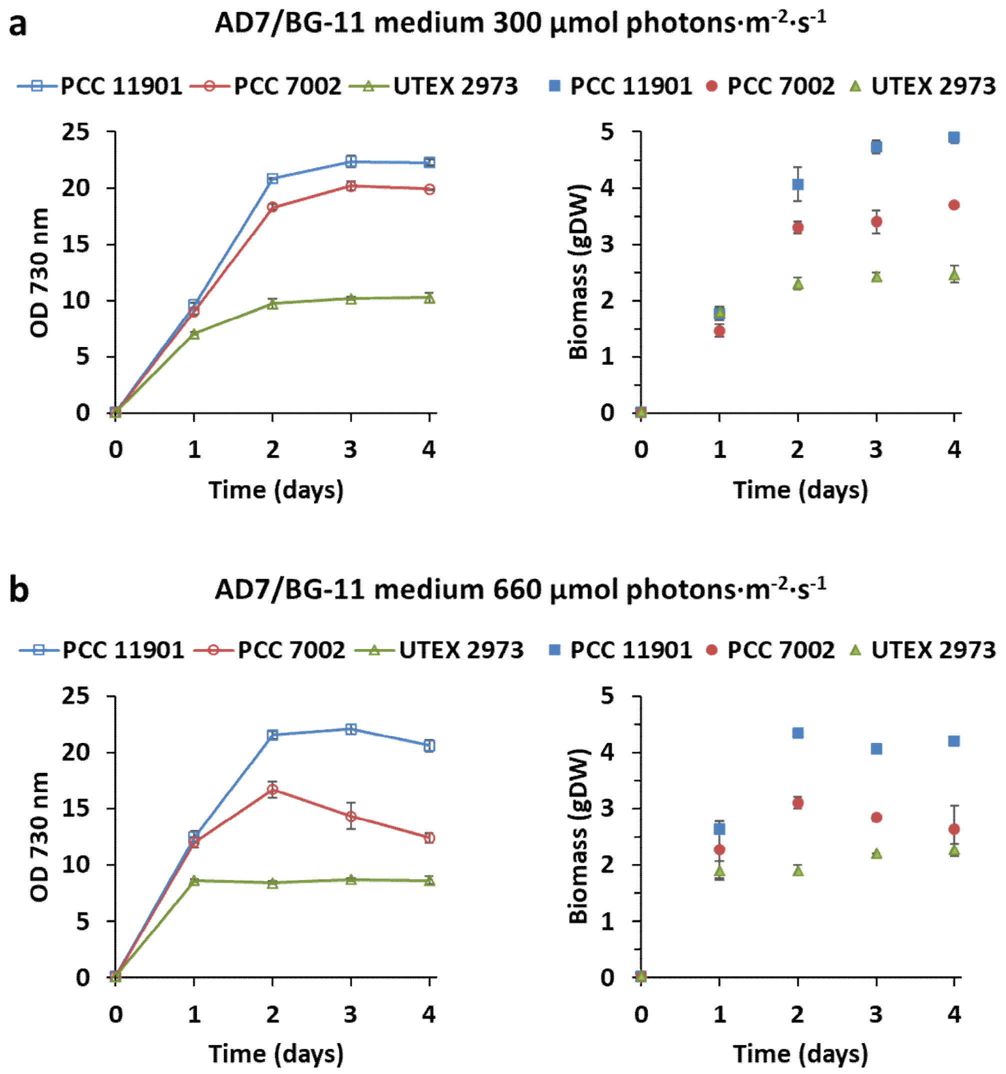

Supplementary Figure 5. Growth performance and biomass accumulation of PCC 11901, PCC 7002 and UTEX 2973 using basic AD7 or BG-11 medium. Lines with empty markers correspond to OD_{730} measurements, whereas filled markers correspond to biomass accumulation. Strains were grown at 38 °C, 225, 1% CO_2 rpm at either **(a)** 300 µmol photons·m^{-2}·s^{-1} RGB 4:2:1 or **(b)** 660 µmol photons·m^{-2}·s^{-1} RB 1:1. Average OD_{730} and biomass were calculated as mean of n=3 biological replicates ± standard deviation.

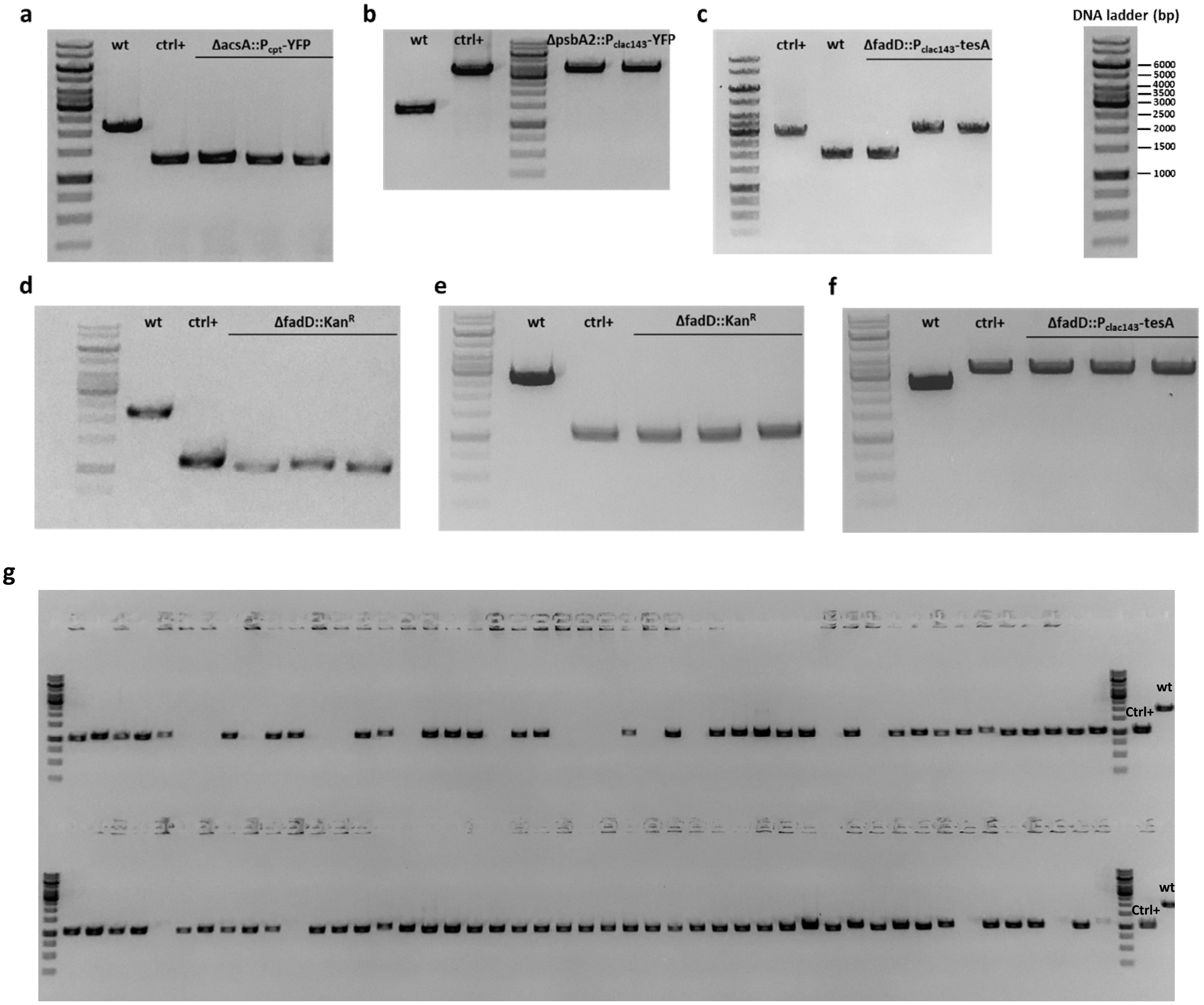

Supplementary Figure 6. Genotyping PCRs of engineered PCC 11901 and PCC 7002 strains. Primers used for the reactions are listed in **Table S4**. Genotyping of **(a)** ΔacsA::P$_{cpt}$-YFP, **(b)** ΔpsbA2::P$_{clac143}$-YFP, **(c)** 11901 ΔfadD::P$_{clac143}$-tesA, **(d)** 11901 ΔfadD::Kan^R, **(e)** 7002 ΔfadD::Kan^R and **(f)** 7002 ΔfadD::P$_{clac143}$-tesA transformants. **(g)** Genotyping of 94 colonies of 11901 ΔfadD::Kan^R for the transformation efficiency assessment. DNA ladder: GeneRuler 1kb (#SM0311, NEB).

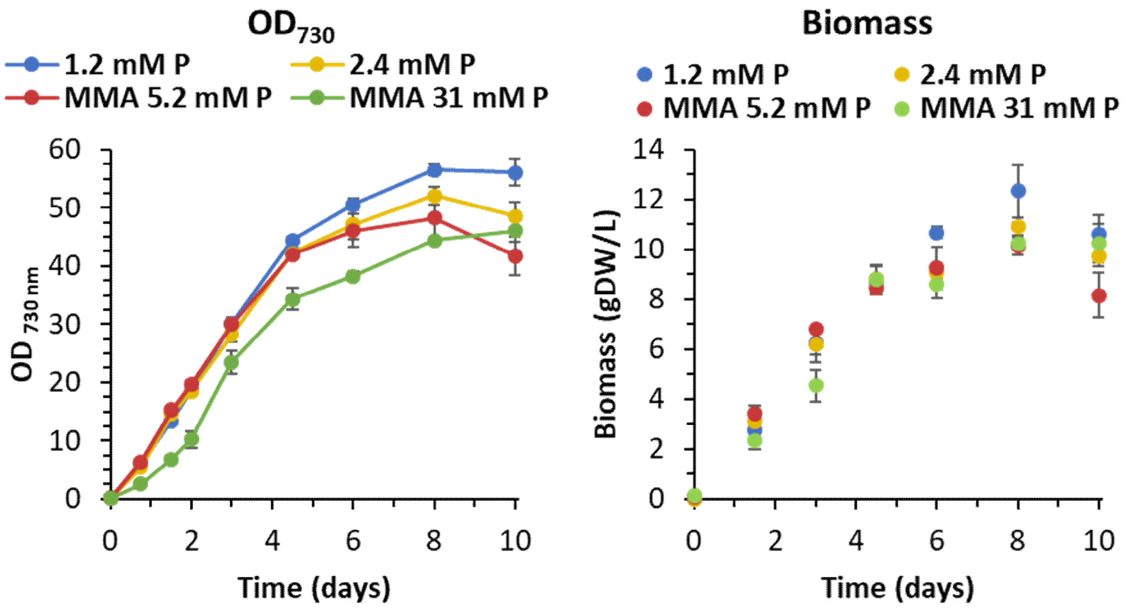

Supplementary Figure 7. Comparison of PCC 11901 strain's growth in different modified media. Two enriched medium formulation variants used in this study were compared with previously published MMA medium optimized for PCC 7002[1]. All cultures were grown in triplicates at 38 °C, 1% CO_2, 200 rpm, 300 µmol photons·m^{-2}·s^{-1}. OD_{730} and dry cell weight were measured in time intervals for 10 days. In blue AD medium supplemented with 96 mM $NaNO_3$, 1.2 mM KH_2PO_4 (P) and 240 µM $FeCl_3$ (MAD), in yellow is the same medium but with more phosphate added (2.4 mM KH_2PO_4). Red and green markers correspond to MMA medium (120 mM $NaNO_3$, 1.1 mM $FeCl_3$) with either 5.2 or 31 mM KH_2PO_4 respectively. Average OD_{730} and biomass were calculated as mean of n=3 biological replicates ± standard deviation.

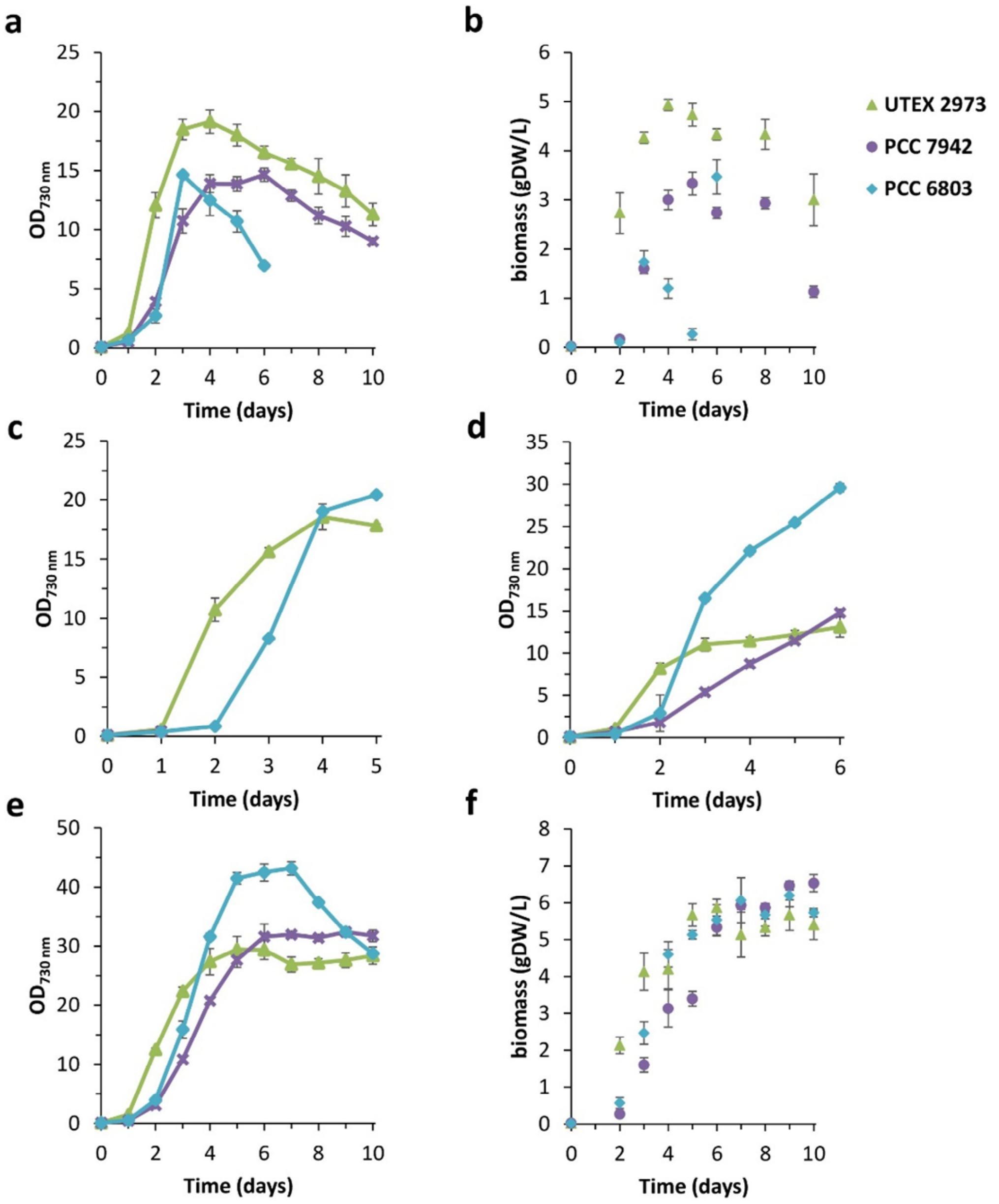

Supplementary Figure 8. Growth and biomass accumulation comparison of UTEX 2973, PCC 7942 and PCC 6803 cultured in different optimized media. All strains were grown at 30 °C, 1% CO_2 and 200 rpm shaking. In the first 24 hours of growth UTEX 2973 was incubated at 150 μmol photons·m^{-2}·s^{-1} light intensity, which was then increased to 750 μmol photons·m^{-2}·s^{-1}. For PCC 7942 and PCC 6803 initial light intensity was set to 75 μmol photons·m^{-2}·s^{-1}, changed to 150 μmol photons·m^{-2}·s^{-1} after 1 day and then increased to 750 μmol photons·m^{-2}·s^{-1} on day 2. RGB ratio of LED lights was set to 1:1:1 throughout whole experiment. **(a,b)** Growth and dry cell weight for all freshwater strains grown in MBG medium (BG-11 with 96 mM $NaNO_3$ and 1.2 mM KH_2PO_4 and 240 μM ammonium iron (III) citrate) **(c)** MBG supplemented with 20 mM $MgSO_4$, **(d)** MAD medium with 0% NaCl and **(e,f)** 5xBGM (5xBG medium with 96 mM $NaNO_3$, 0.6 mM KH_2PO_4 and 0.6 mM K_2HPO_4). All above media recipes are listed in **Table S3**. Average OD_{730} and biomass were calculated as mean of n=3 biological replicates ± standard deviation.

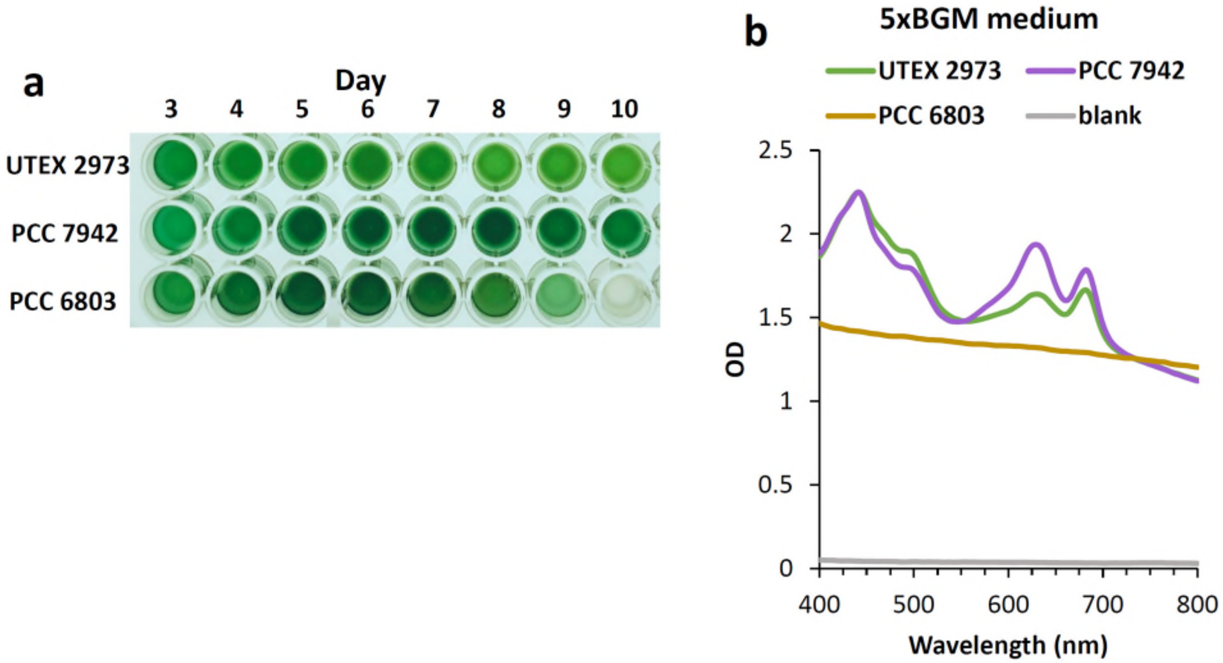

Supplementary Figure 9. Pigmentation of UTEX 2973, PCC 7942 and PCC 6803 cultures in 5xBGM medium. **(a)** Appearance of all strains grown at 30 °C, 200 rpm, 1% CO_2 for 10 days. **(b)** Whole cell spectra of cultures collected after 10 days of continuous growth.

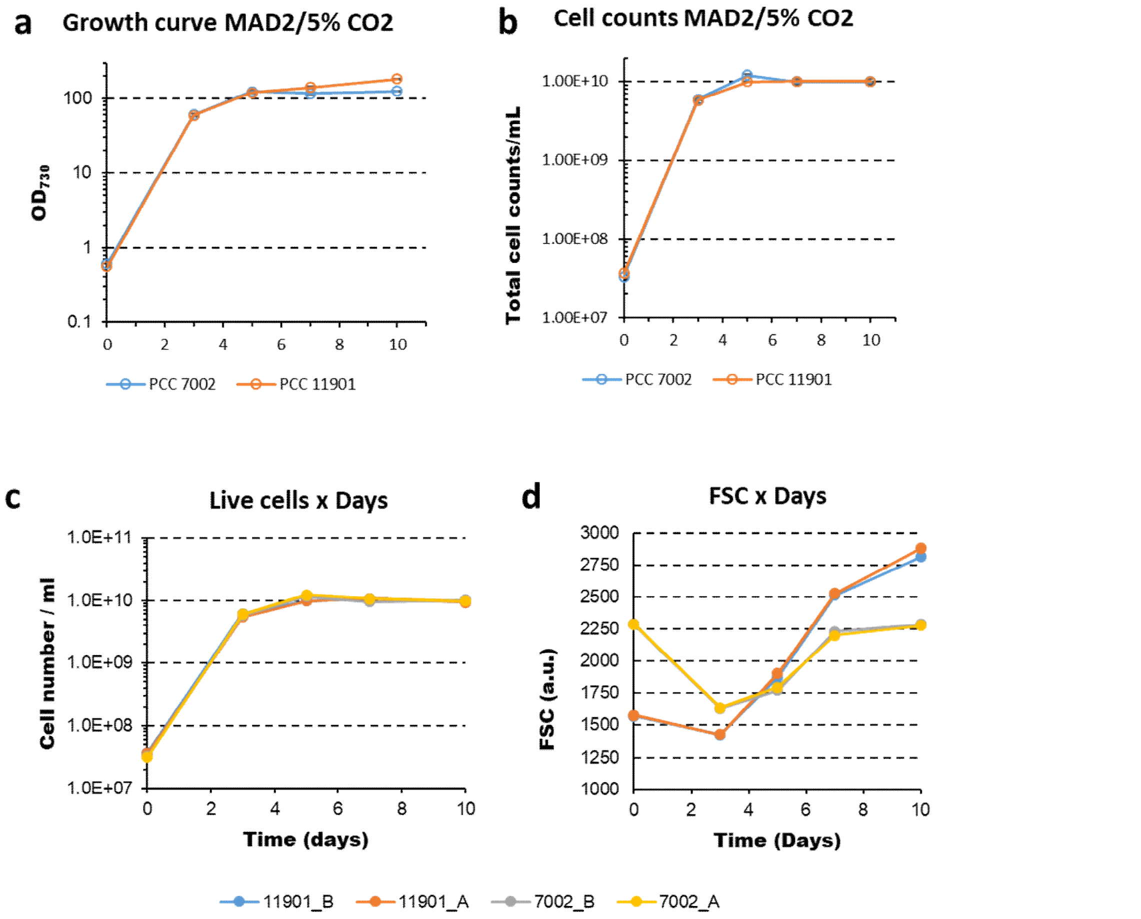

Supplementary Figure 10. Graphical representation of OD_{730} **(a)**, total cell counts/mL **(b)**, live cell counts/mL **(c)** and forward scatter (FSC) **(d)** variation over time for PCC 7002 and PCC 11901 cultures. Data shown is for two independent cultures per strain. Variation at day 10 is statistically significant for **a** (p=0.00059) and **d** (p=0.0037).

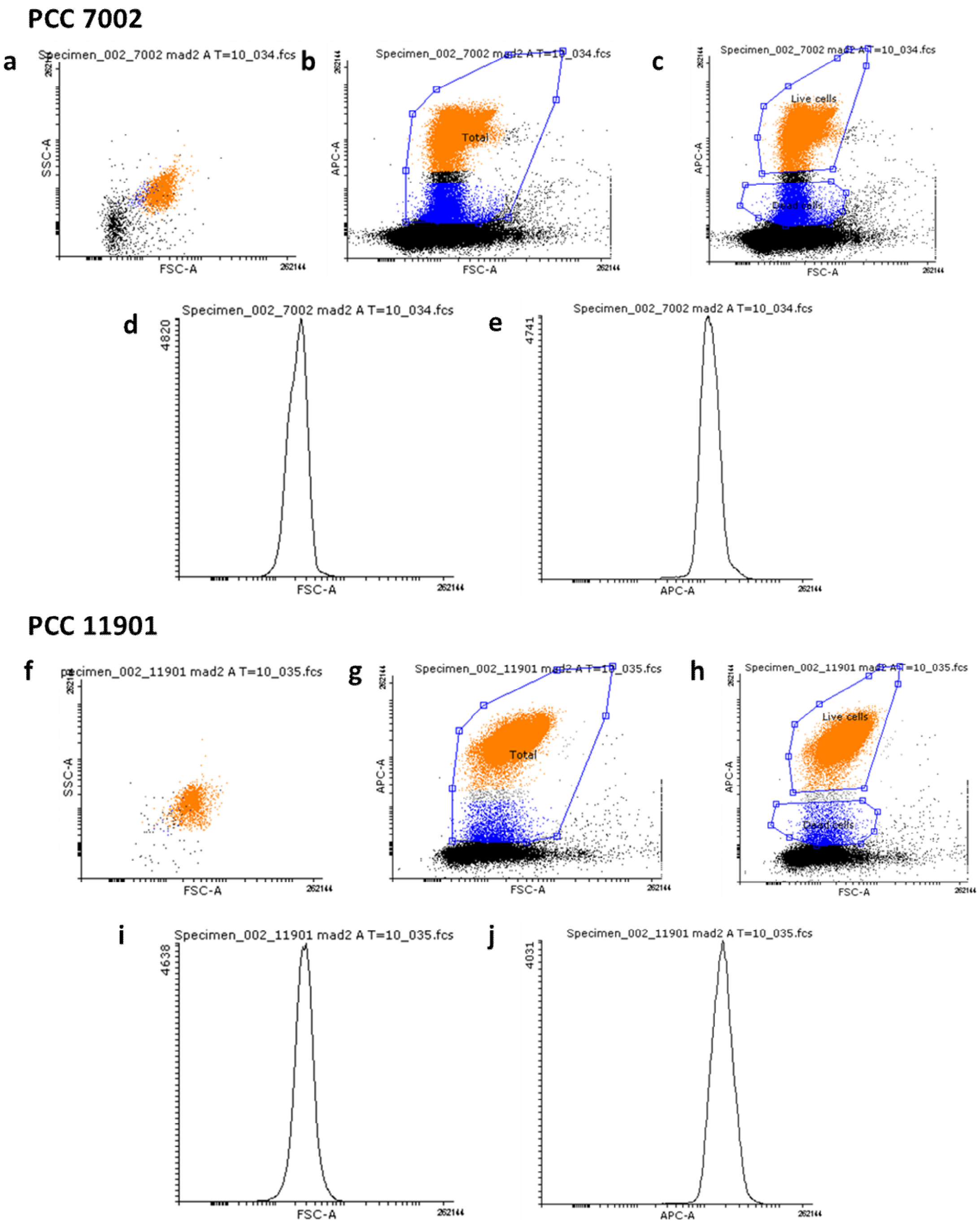

Supplementary Figure 11. Gating strategy used for flow cytometry-based cell count and cell size estimation experiments. Data shown is for the 10 days data point. **(a, f)** Side scatter (SSC) vs forward scatter (FSC). Gates drawn on dot plots for same populations as in **a** and **f**, using allophycocyanin (APC-A) vs FSC for whole population **(b, g)** or live vs dead cell populations **(c, h)**. **(d, i)** Histogram of FSC based on whole cell population dot plots. **(e, j)** Histogram of APC-A based on whole cell population.

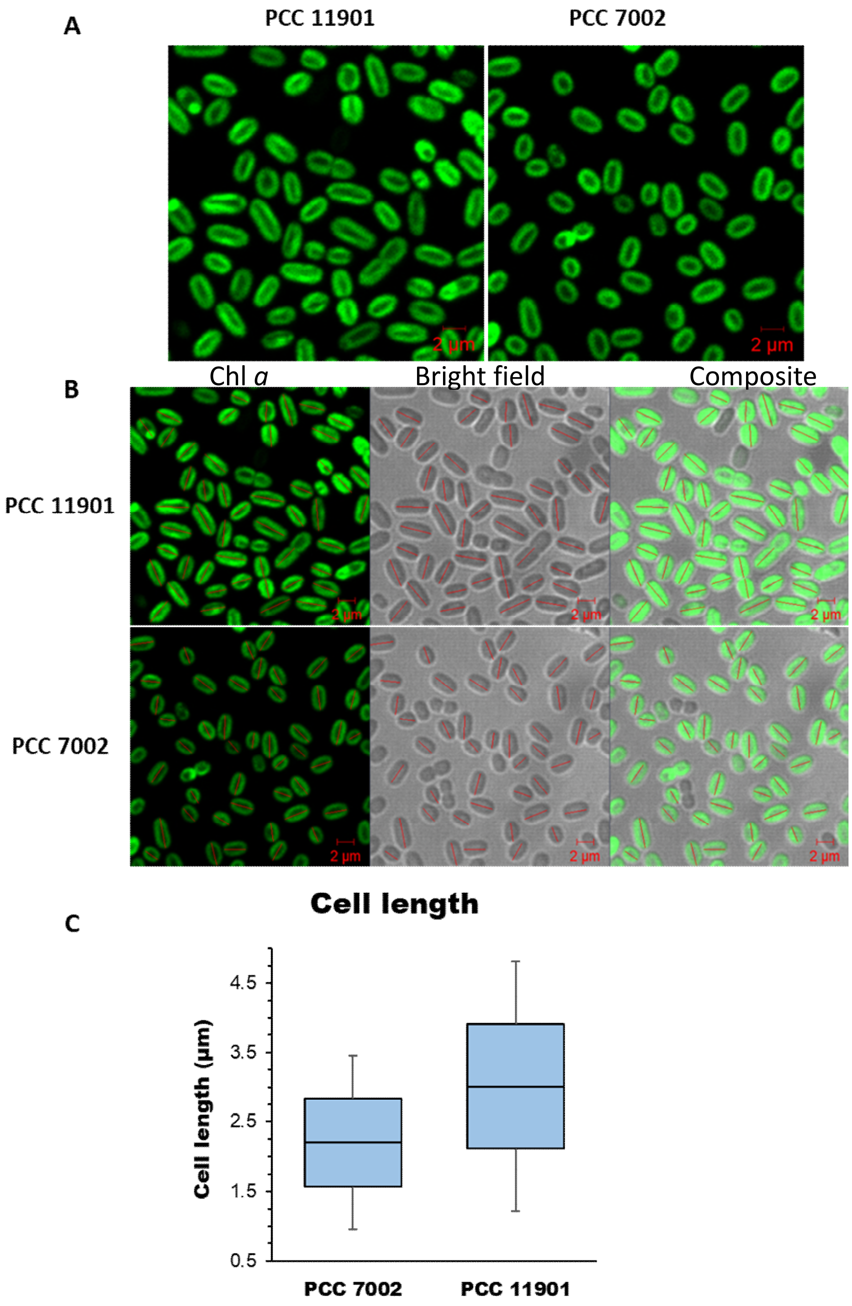

Supplementary Figure 12. (a) Confocal fluorescence microscopy images of PCC 7002 and PCC 11901 at day 10. **(b)** Micrographs with overlays for cell length determination. **(c)** Box and whisker plots for PCC 7002 and PCC 11901 cell length distribution. Data shown is for n=102 cells, at 10 days post-inoculation in MAD2 medium, 5% CO_2 and 700 µmol photons·m^{-2}·s^{-1}. Variation is statistically significant (ANOVA, $p=5\cdot10^{-23}$, $\eta^2=0.621$).

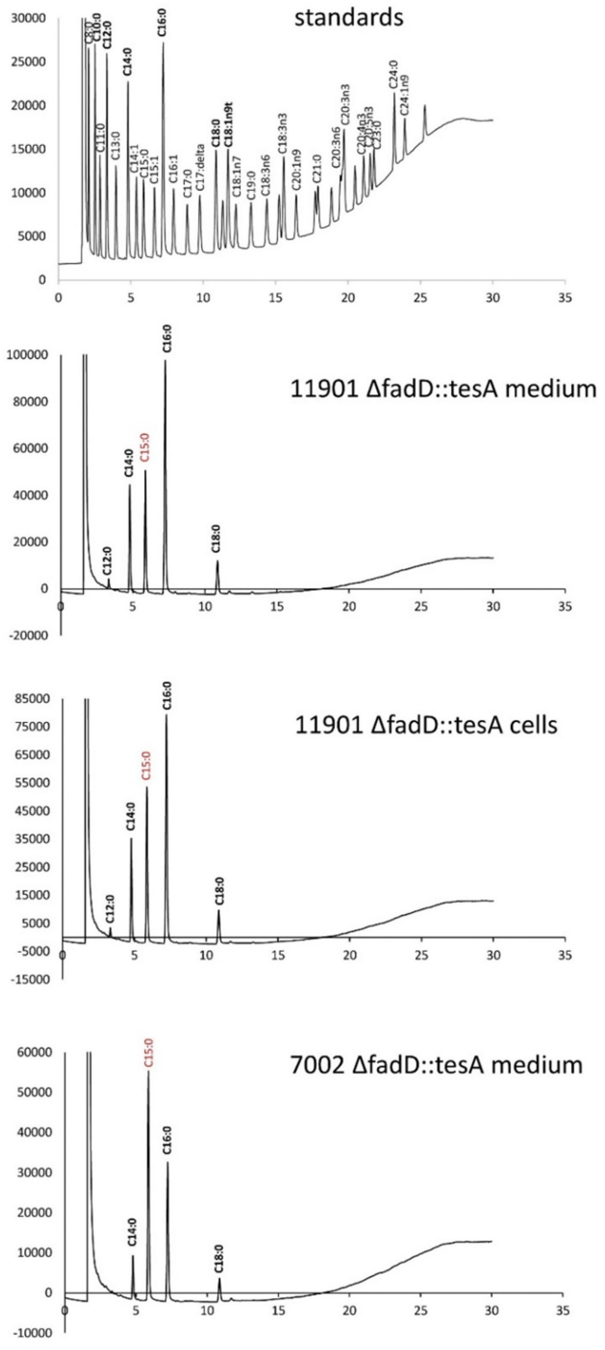

Supplementary Figure 13. GC chromatograms of medium and cell extracts samples of engineered FFA producing strains. Samples were spiked with internal C15:0 standard.

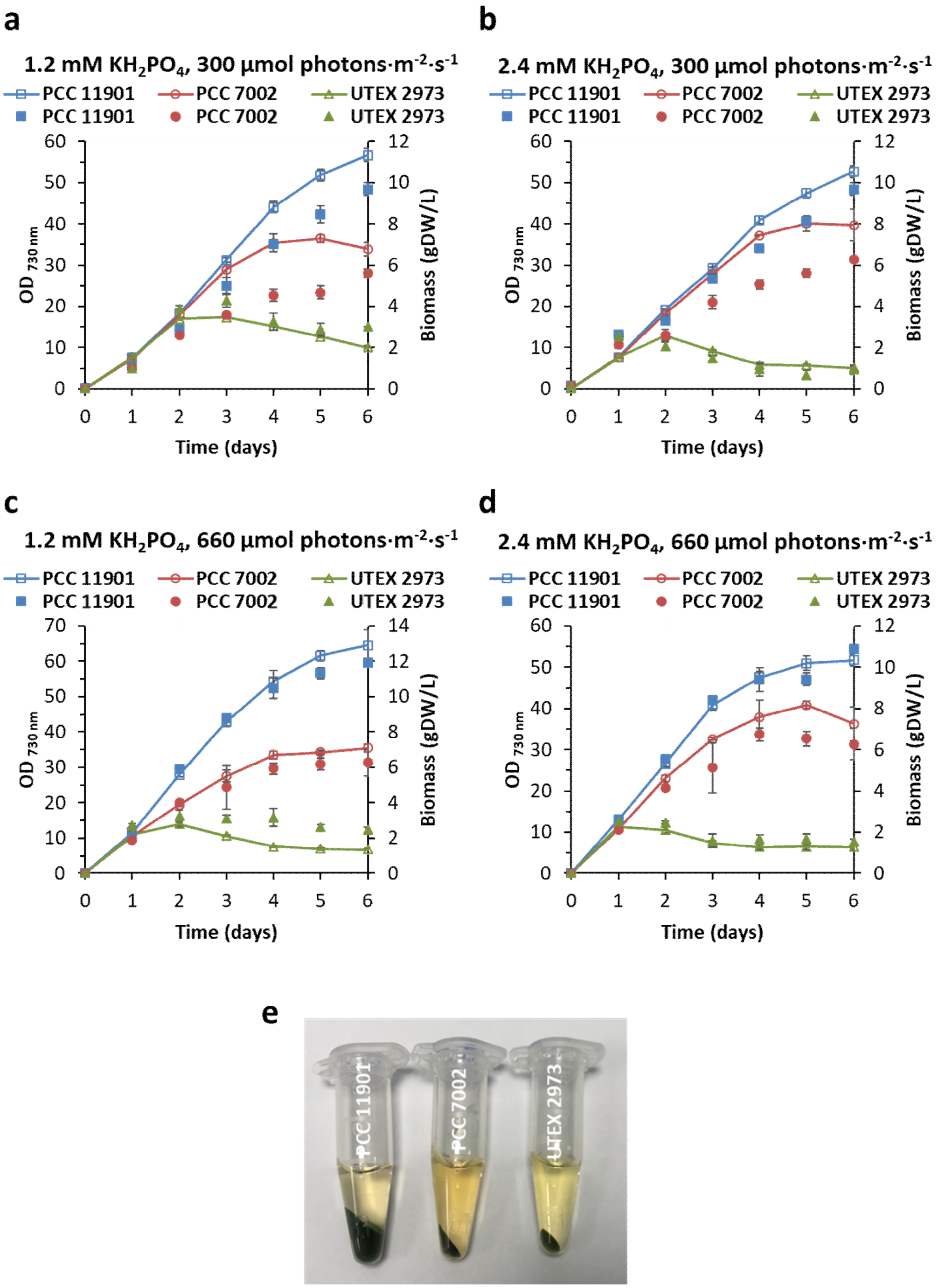

Supplementary Figure 14. Growth performance and biomass accumulation of PCC 11901, PCC 7002 and UTEX 2973 using optimized MAD and MBG media. Lines with empty markers correspond to OD_{730} measurements, whereas filled markers correspond to biomass accumulation. Strains were grown at 38 °C, 1% CO_2, 200 rpm at constant illumination of either 300 µmol photons·m^{-2}·s^{-1} RGB 4:2:1 **(a, b)** or 660 µmol photons·m^{-2}·s^{-1} RB 1:1 **(c, d)**. MAD and MBG media used for cultivation of strains contained either 1.2 mM **(a,c)** or 2.4 mM **(b, d)** of KH_2PO_4. Average OD_{730} and biomass were calculated as mean of n=3 biological replicates ± standard deviation. **(e)** Display of cell pellets centrifuged using 0.5 mL of cultures after 6 days of cultivation.

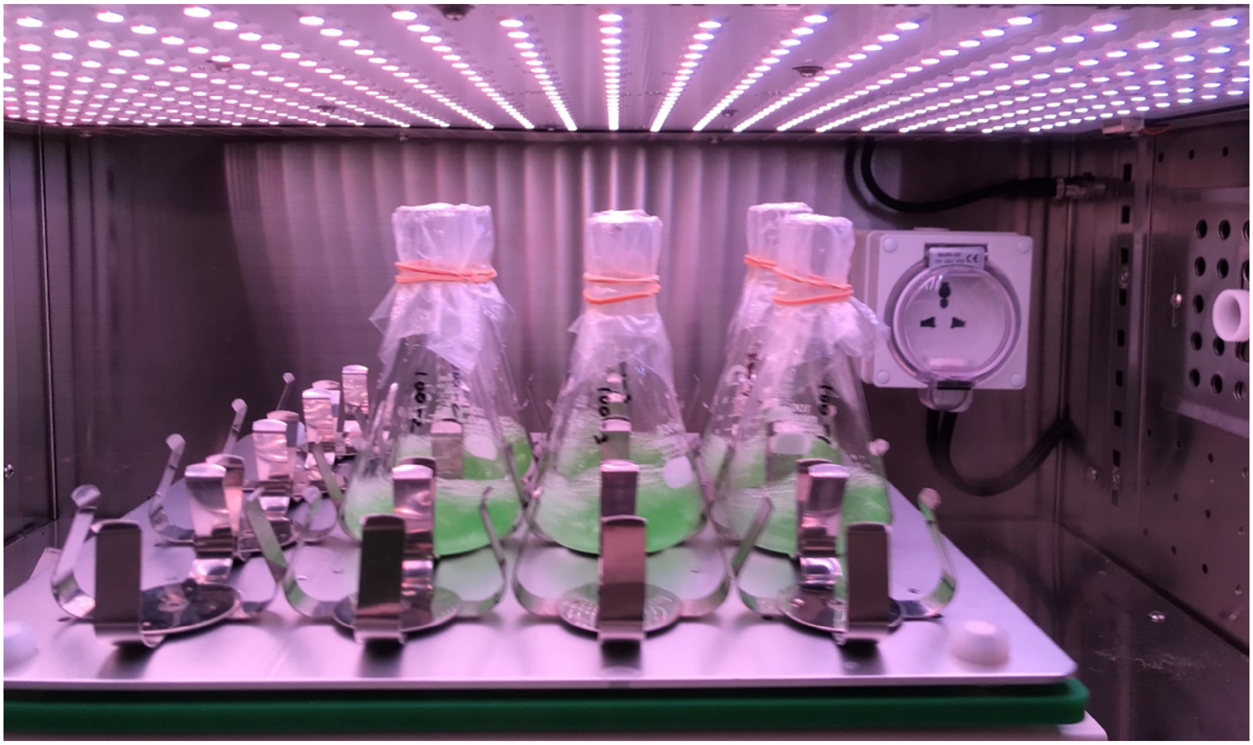

Supplementary Figure 15. Cyanobacterial growth facility setup.

Supplementary Tables

Supplementary Table 1. Doubling times of *Synechococcus* sp. PCC 11901, *Synechococcus* sp. PCC 7002 and *Synechococcus elongatus* UTEX 2973. Strains were grown side by side under different conditions. For all lighting conditions RGB ratio was set to 4:2:1 except for 660*, where the red and blue light were used only at 1:1 ratio. All doubling times are given in hours. Average doubling time was calculated as mean of n=3 biological replicates ± standard deviation.

	Doubling times in different conditions (h)											
	0.04% CO_2		1% CO_2									
**Temp. (°C)**	**38**		**30**	**34**		**38**			**41**		**43**	
**Light intensity (µmol photons·m^{-2}·s^{-1})**	**100**	**300**	**300**	**300**	**500**	**300**	**500**	**660***	**300**	**500**	**100**	**300**
**PCC 11901**	3.35 ± 0.12	3.85 ± 0.12	3.53 ± 0.03	2.92 ± 0.07	3.02 ± 0.04	2.46 ± 0.02	2.33 ± 0.02	2.14 ± 0.06	2.62 ± 0.07	2.80 ± 0.08	3.32 ± 0.16	2.69 ± 0.06
**PCC 7002**	3.59 ± 0.12	3.96 ± 0.22	3.19 ± 0.01	n/a	n/a	2.46 ± 0.09	2.27 ± 0.01	2.29 ± 0.13	2.55 ± 0.13	2.83 ± 0.16	n/a	n/a
**UTEX 2973**	3.65 ± 0.32	3.05 ± 0.05	3.33 ± 0.02	n/a	n/a	2.08 ± 0.06	2.02 ± 0.01	2.02 ± 0.04	2.15 ± 0.05	1.93 ± 0.04	n/a	n/a

Supplementary Table 2. List of predicted proteins present in genome major insertions of *Synechococcus* sp. PCC 11901 found by BLAST search analysis.

Locus tag	Protein name (highest identity)
FEK30_11785	ABC transporter permease [Synechococcus sp. NKBG042902]
FEK30_11790	ABC transporter ATP-binding protein [Synechococcus sp. NKBG042902]
FEK30_11795	acyltransferase [Synechococcus sp. NKBG042902]
FEK30_11800	FkbM family methyltransferase [Synechococcus sp. NKBG042902]
FEK30_11805	glycosyltransferase family 4 protein [Synechococcus sp. NKBG042902]
FEK30_11810	glycosyltransferase [Synechococcus sp. NKBG042902]
FEK30_11815	glycosyltransferase family 2 protein [Nodularia sp. NIES-3585]
FEK30_11820	glycosyltransferase [Nostoc sp. NIES-3756]
FEK30_11825	hypothetical protein [Nodularia sp. NIES-3585]
FEK30_11830	glycosyltransferase [Nodularia sp. NIES-3585]
FEK30_11835	glycosyltransferase family 2 protein [filamentous cyanobacterium CCP2]
FEK30_11840	glycosyltransferase [Nostoc sp. PCC 7524]
FEK30_11845	glycosyltransferase family 2 protein [Synechococcus sp. NKBG042902]
FEK30_11850	acyltransferase [Synechococcus sp. NKBG042902]
FEK30_11855	glycosyltransferase family 2 protein [Nostoc sp. NIES-3756]
FEK30_11860	glycosyltransferase family 4 protein [Synechococcus sp. NKBG042902]
FEK30_11865	glycosyltransferase family 2 protein [Synechococcus sp. NKBG042902]
FEK30_11870	glycosyltransferase family 4 protein [Synechococcus sp. NKBG042902]
FEK30_11875	glycosyltransferase family 4 protein [Synechococcus sp. NKBG042902]
FEK30_11880	hypothetical protein [Synechococcus sp. NKBG042902]
FEK30_11885	glycosyltransferase [Synechococcus sp. NKBG042902]
FEK30_12515	IS5 family transposase [Synechococcus sp. NKBG042902]
FEK30_12765	DGQHR domain-containing protein [Nostocales cyanobacterium]
FEK30_13300	DUF3854 domain-containing protein [Synechococcus sp. NKBG042902]
FEK30_13890	phospholipid carrier-dependent glycosyltransferase [Synechococcus sp. PCC 7003]
FEK30_15055	hypothetical protein [Trichormus sp. NMC-1]
FEK30_15475	IS256 family transposase [Synechococcus sp. NKBG042902]
FEK30_15980	F0F1 ATP synthase subunit beta [Synechococcus sp. NKBG042902]
FEK30_15515	IS5 family transposase [Synechococcus sp. NKBG042902]
FEK30_15850	IS5 family transposase [Synechococcus sp. NKBG042902]
FEK30_01650	NAD(P)-dependent alcohol dehydrogenase [Synechococcus sp. NKBG042902]
FEK30_01865	DNA cytosine methyltransferase [Coleofasciculus chthonoplastes]
FEK30_01875	helix-turn-helix transcriptional regulator [Chroococcidiopsis sp. CCALA 051]
FEK30_03655	BrnT family toxin [Anabaenopsis circularis]
FEK30_06150	TPA: methicillin resistance protein [Syntrophomonas sp.]
FEK30_06155	MULTISPECIES: methionyl-tRNA formyltransferase [Vibrio]
FEK30_06165	translocase [Anaerolineaceae bacterium 4572_78]
FEK30_09280	type II toxin-antitoxin system RelB/DinJ family antitoxin [Synechococcus sp. BDU 130192]

FEK30_09285	type II toxin-antitoxin system YafQ family toxin
FEK30_09390	MULTISPECIES: Fic family protein [Synechococcus]
FEK30_09715	IS5 family transposase [Synechococcus sp. NKBG042902]
FEK30_09720	MULTISPECIES: IS982 family transposase [unclassified Cyanobacteria (miscellaneous)]
FEK30_10375	IS630 family transposase [Cyanothece sp. PCC 7425]
FEK30_10580	MULTISPECIES: IS982 family transposase [unclassified Cyanobacteria (miscellaneous)]
FEK30_11580	MULTISPECIES: S-layer homology domain-containing protein [Synechococcus]

Supplementary Table 3. Media formulations used for the growth experiments[#]. **All concentrations are in mM**. Media in which marine (MAD) and freshwater (5xBG) cyanobacterial strains performed best were marked with star (*). (Note: The protocol for medium preparation is available on the last page of this supplementary material)

	AD7	BG-11	MAD*	MAD2	MBG	MBG-Mg+	5xBG*	5xBGM
**$NaNO_3$**	12	17.6	96	192	96	96	88	96
**KH_2PO_4**	0.37	-	1.2	2.4	-	-	-	0.6
**K_2HPO_4**	-	0.175	-	-	1.2	1.2	0.875	0.6
**NaCl**	308	-	308	308	-	-	-	-
**KCl**	8	-	8	8	-	-	-	-
**$CaCl_2 \cdot 2H_2O$**	2.5	0.245	2.5	2.5	0.245	0.245	1.22	1.22
**Na_2EDTA**	0.081	0.027	0.081	0.081	0.027	0.027	0.13	0.13
**$MgSO_4 \cdot 7H_2O$**	20.3	0.3	20.3	20.3	0.3	20.3	1.52	1.52
**$FeCl_3 \cdot 6H_2O$**	0.015	-	0.24	0.48	-	-	-	-
**Ammonium iron (III) citrate**	-	0.023	-	-	0.24	0.24	0.24	0.24
**Tris-HCl**	8.6	-	8.6	8.6	-	-	-	-
**H_3BO_3**	0.046	0.046	0.046	0.138	0.046	0.046	0.231	0.231
**$MnCl_2 \cdot 4H_2O$**	0.0091	0.0091	0.0091	0.0273	0.0091	0.0091	0.0457	0.0457
**$ZnSO_4 \cdot 7H_2O$**	0.00077	0.00077	0.00077	0.00213	0.00077	0.00077	0.00386	0.00386
**$Na_2MoO_4 \cdot 2H_2O$**	0.00521	0.00161	0.00521	0.01563	0.00161	0.00161	0.00806	0.00806
**$CuSO_4 \cdot 5H_2O$**	0.00032	0.00032	0.00032	0.00096	0.00032	0.00032	0.00158	0.00158
**$(CoNO_3)_2 \cdot 6H_2O$**	-	0.00017	-	-	0.00017	0.00017	0.00084	0.00084
**$CoCl_2 \cdot 6H_2O$**	0.00017	-	0.00017	0.00051	-	-	-	-
**vitamin B_{12}**	0.003	-	0.003	0.009	-	-	-	-
**Na_2CO_3**	-	0.189	-	-	0.189	0.189	0.472	0.943
**citric acid**	-	0.031	-	-	0.031	0.031	0.156	0.156

[#]During MAD and MAD2 medium preparation excessive phosphate was added as the last component. At this stage medium can be either filtered using 0.22 µm sterile filters (no precipitate forming) or autoclaved (precipitate of phosphate salts will form). 5xBG and 5xBGM media were sterile-filtered.

[##]2000x concentrated and filter sterile $FeCl_3$ stocks were prepared in 0.1 M HCl and added to the medium only upon medium filtering or autoclaving to avoid formation of poorly soluble iron hydroxides.

Supplementary Table 4. List of vectors and primers used for the amplification of DNA blocks for the Gibson assembly. (*) – plasmids obtained from other labs (as specified in the Methods section).

Plasmid name	PCR template	PCR product	Primer name	Primer sequence (5'-3')
pSW036 (Addgene ID: 140034)	Synechococcus sp. PCC 11901 gDNA	acsA upstream FR	SSW07_acsA_up_F	GACGTTGTAAAACGACGGCCAGTgcaatgctgagatgatcctcggtgaa
			SSW07_acsA_up_R	ttattatcgtgggatttatttcaccccatt
	pAcsA_cpt_YFP*	Pcpt-YFP	cpt-YFP_acsA_F	aatggggtgaaataaatcccacgataataattaacaaaaaagcaggaataaaattaacaagatgtaac
			cpt-YFP_acsA_R	aagacaccctctgtcctctggacatctttgaggccgttgatctagacaaa
	Synechococcus sp. PCC 11901 gDNA	acsA downstream FR	SSW07_acsA_dw_F	agatgtccagaggacagagggtgt
			SSW07_acsA_dw_R	CAATTTCACACAGGAAACAGCTATGACagtaaatcagagacagaaaccttccagacg
	pUC19	pUC backbone	pUC19_B_F	GTCATAGCTGTTTCCTGTGTGAAATTGTTATC
			pUC19_B_R	ACTGGCCGTCGTTTTACAACGT
pSW039 (Addgene ID: 140035)	Synechococcus sp. PCC 11901 gDNA	psbA2 upstream FR	SSW07_psbA2_up_F	CAGTCACGACGTTGTAAAACGACGGCCAGTaattagtgtaggagatcaccgttgaattgatcac
			SSW07_psbA2_up_R	aattgcatgtaccagtttaaagtactgggg
	pAcsA_cLac143_YFP*	Pclac143-YFP	clac143-YFP_psbA2_F	ccccagtactttaaactggtacatgcaattgactcccctctggacatctcca
			clac143-YFP_psbA2_R	aaccacacgcacaattcccttaaaaagcaacattaattgcgttgcgctcactg
	Synechococcus sp. PCC 11901 gDNA	psbA2 downstream FR	SpR_psbA2_F	tgctttttaagggaattgtgcgtgtggtt
			SpR_psbA2_R	ccactgcaatcccaaattcaaacttccagAAACGGATGAAGGCACGAACCCAG
	pDF-trc*	$Spec^R$	SSW07_psbA2_dw_F	ctggaagtttgaatttgggattgcagtgg
			SSW07_psbA2_dw_R	TTTCACACAGGAAACAGCTATGACtggcaggcgagacaactgctttcg
	pUC19	pUC backbone	pUC19_B_F	GTCATAGCTGTTTCCTGTGTGAAATTGTTATC
			pUC19_B_R	ACTGGCCGTCGTTTTACAACGT
pSZ013 (used for subcloning only)	codon optimized tesA from GenScript	tesA	ICA_tesA_to_cLac_F	gataacaatttcacacaccaactcataaagtcaagtaggagattaattccATGGATACCCTCCTCATTCTC
			ICA_tesA_to_cLac_R	gtggcagcagccaactcagcttcctttcgggctttgttagacagccggatAGGGCCGAGTTTGTACAAG
	pAcsA_cLac143_YFP	pAcsA_cLac143_YFP backbone	pAcsA_bef_pMB2_F	ATCCGGCTGTCTAACAAAG
			pAcsA_lacO_R	GGAATTAATCTCCTACTTGACTTTATG
pUC19-fadD (used for subcloning only)	codon optimized tesA from GenScript	tesA	InF_fadD_F	ACCCGGGGATCCTCTTCAGGTCAATGACATTGC
			InF_fadD_R	CTGCAGGTCGACTCTACCAGATTATCGCCCACTTTC
	pUC19	puc19 backbone	XbaI digested	
pSZT025 (Addgene ID: 140033)	pUC57-kan	Kan^R	ICA_KanR_F	gggatctcgaccgatgcccttgagaATTAAGGAGTGGACAacattgc
			ICA_KanR_R	TCCGCCATTCGGATCGCTTAATCTACGTGAACCAAGTCATTAGAAAAAC
	pUC19	puc19	ICA_open_fadD_F	gtttttctaaTGACTTGGTTCACGTAGATTAAGCGA

		backbone	ICA_open_fadD_R	TCCGAATGG<br>CACAATTCGtttggagatgtccagaAATTCCATTTAAAAGCGCAAAAAAC
pSW040 (used for subcloning only)	Synechococcus sp. PCC 11901 gDNA	fadD FR	SSW fadD up F2<br>SSW07 fadD dw R2	GTAACGCCAGGGTTTTCCCAGTCACGACGTTGTAAAACGACGGCCAGTtaattaaaatttgactgcgatcgcctgcaatggcctgggcctgggcgacaat<br>ATGTTGTGTGGAATTGTGAGCGGATAACAATTTCACACAGGAAACAGCTATGACaaatcgccgtggatttgaggtggggaataagggtttgagccgtg
	pUC19	pUC backbone	pUC19_B_F<br>pUC19_B_R	GTCATAGCTGTTTCCTGTGTGAAATTGTTATC<br>ACTGGCCGTCGTTTTACAACGT
pSW068 (Addgene ID: 140036)	pSZ025	clac143-tesA	clac143-fadD F<br>clac143-fadD R	cttttaaatggaattgcctctggacatctccaaaCGAATTGTGA<br>gatatatttttatcttgtgcaatgtTAATCTagAAAGATGACTAATTCGCTCGTC
	pSW040	pUC-fadD backbone	pUC19_B_F<br>pUC19_B_R	GTCATAGCTGTTTCCTGTGTGAAATTGTTATC<br>ACTGGCCGTCGTTTTACAACGT
pSW071 (Addgene ID: 140037)	pSW068	pSW068 w/o tesA	SSW fadD KO F<br>SSW fadD KO R	tggaattgccacattgcacaagataaaaatatatcatcatgaacaataaaact<br>caatgtggcaattccatttaaaagcgcaaaaaacaag
pSW072	pSZ025	pSZ025 w/o tesA	SSW fadD KO F<br>SSW fadD KO R	tggaattgccacattgcacaagataaaaatatatcatcatgaacaataaaact<br>caatgtggcaattccatttaaaagcgcaaaaaacaag

Supplementary Table 5. List of primers used for sequencing of constructs and genotyping of transformed cyanobacterial strains.

Primer name	Primer sequence (5'-3')	Binding region
CYA361f	GGAATTTTCCGCAATGGG	Cyanobacterial 16S rRNA
CYA785r	GACTACWGGGGTATCTAATCC	Cyanobacterial 16S rRNA
27f	AGAGTTTGATCCTGGCTCAG	Bacterial 16S rRNA
1492r	TACCTTGTTACGACTT	Bacterial 16S rRNA
SSW_seg_acsA_F	aggtcatatccgaggcgtacattca	PCC 11901 *acsA* upstream
SSW_seg_acsA_R	gaactaggtcaaggcaagcagtcgg	PCC 11901 *acsA* downstream
SSW07 psbA2 seg F	gtgattaagcaataaatcgattgagcga	PCC 11901 *psbA2* upstream
SSW07 psbA2 seg R	tcaggattcagagcaaaccaagatta	PCC 11901 *psbA2* downstream
fadD SSW07 seg F	agtaggattgtagccatgatttcgg	PCC 11901 *fadD* upstream
fadD SSW07 seg R	taggcacttggtttccgtccat	PCC 11901 *fadD* downstream

Supplementary Notes

16S rRNA sequence of the isolated bacterial contaminant:

GGCAGCTACCATGCAGTCGAGCGCACCTTCGGGTGAGCGGCGGACGGGTTAGTAACGCGTGGGAACGTACCCTT
TTCTAAGGAATAGCCACTGGAAACGGTGAGTAATACCTTATACGCCCTTCGGGGGAAAGATTTATCGGAGAAGGA
TCGGCCCGCGTTAGATTAGATAGTTGGTGGGGTAACGGCCTACCAAGTCTACGATCTATAGCTGGTTTTAGAGGAT
GATCAGCAACACTGGGACTGAGACACGGCCCAGACTCCTACGGGAGGCAGCAGTGGGGAATCTTGGACAATGGG
CGCAAGCCTGATCCAGCCATGCCGCGTGAGTGATGAAGGCCTTAGGGTCGTAAAGCTCTTTCGCTGGGGATGATA
ATGACAGTACCCAGTAAAGAAACCCCGGCTAACTCCGTGCCAGCAGCCGCGGTAATACGGAGGGGGTTAGCGTT
GTTCGGAATTACTGGGCGTAAAGCGCGCGTAGGCGGATTGGAAAGTTGGGGGTGAAATCCCGGGGCTCAACCTC
GGAACTGCCTCCAAAACTATCAGTCTAGAGTTCGAGAGAGGTGAGTGGAATTCCGAGTGTAGAGGTGAAATTCGT
AGATATTCGGAGGAACACCAGTGGCGAAGGCGGCTCACTGGCTCGATACTGACGCTGAGGTGCGAAAGTGTGGG
GAGCAAACAGGATTAGATACCCTGGTAGTCCACACCGTAAACGATGAATGCCAGTCGTCAGCAAGCATGCTTGTT
GGTGACACACCTAACGGATTAAGCATTCCGCCTGGGGAGTACGGTCGCAAGATTAAAACTCAAAGGAATTGACGG
GGGCCCGCACAAGCGGTGGAGCATGTGGTTTAATTCGAAGCAACGCGCAGAACCTTACCAACCCTTGACATCCTG
TGCTACATCCAGAGATGGATGGTTCCCTTCGGGGACGCAGTGACAGGTGCTGCATGGCTGTCGTCAGCTCGTGTC
GTGAGATGTTCGGTTAAGTCCGGCAACGAGCGCAACCCACATCTTCAGTTGCCAGCAGTTCGGCTGGGCACTCTG
GAGAAACTGCCCGTGATAAGCGGGAGGAAGGTGTGGATGACGTCAAGTCCTCATGGCCCTTACGGGTTGGGCTA
CACACGTGCTACAATGGCAGTGACAATGGGTTAATCCCCAAAAACTGTCTCAGTTCGGATTGTTCTCTGCAACTCG
AGAGCATGAAGTCGGAATCGCTAGTAATCGCGTAACAGCATGACGCGGTGAATACGTTCCCGGGCCTTGTACACA
CCGCCCGTCACACCATGGGAGTTGGGTTTACCCGAAGACGGTGCGCCAACCTTTAGGAGGCAGCTGCCACGTANT
ANNNNNNNNCCGGCTAACTCCGTGCCAGCAGCCGCGGTAATACGGAGGGGGTTAGCGTTGTTCGGAATTACTG
GGCGTAAAGCGCGCGTAGGCGGATTGGAAAGTTGGGGGTGAAATCCCGGGGCTCAACCTCGGAACTGCCTCCAA
AACTATCAGTCTAGAGTTCGAGAGAGGTGAGTGGAATTCCGAGTGTAGAGGTGAAATTCGTAGATATTCGGAGG
AACACCAGTGGCGAAGGCGGCTCACTGGCTCGATACTGACGCTGAGGTGCGAAAGTGTGGGGAGCAAACAGGAT
TAGATACCCTGGTAGTCCACACCGTAAACGATGAATGCCAGTCGTCAGCAAGCATGCTTGTTGGTGACACACCTAA
CGGATTAAGCATTCCGCCTGGGGAGTACGGTCGCAAGATTAAAACTCAAAGGAATTGACGGGGGCCCGCACAAG
CGGTGGAGCATGTGGTTTAATTCGAAGCAACGCGCAGAACCTTACCAACCCTTGACATCCTGTGCTACATCCAGAG
ATGGATGGTTCCCTTCGGGGACGCAGTGACAGGTGCTGCATGGCTGTCGTCAGCTCGTGTCGTGAGATGTTCGGT
TAAGTCCGGCAACGAACGCAACCCACATCTTCAGTTGCCAGCAGTTCGGCTGGGCACTCTGGAGAAACTGCCCGT
GATAAACCGGAAGGAAGGTGTTGAATGACGTCAAGTCCTCCAGGGCCCTTACCGGGTTGGGGTTACCACCGTGGC
TACAATGGCCAGTGACAATGGGGTTAATCCCCCAAAAACTGTTCCCAGTTCCGAATTGTTTCCTTGCAACTCGGAG
AGCTTGAAATCCGGAA

Supplementary Methods

AD7 medium (1 L) preparation protocol:

NaCl	18 g
$MgSO_4 \cdot 7H_2O$	5 g
A^+ modified 100x	10 mL
Tris (125 g/L), pH=8.2	8.3 mL
Modified D7 1000x	1 mL

Autoclave (or sterilize using 0.22 µm filter) and cool down to room temperature to add:

$FeCl_3 \cdot 6H_2O$ (30 mM)	0.5 mL
Vitamin B_{12}	1 mL

For plates, add 12 g/L bacto-agar and 2 g/L of sodium thiosulfate.

Stock solutions:

Modified D7 metal 1000x

	**1 L**
H_3BO_3	2.86 g
$MnCl_2 \cdot 4H_2O$	1.81 g
$ZnSO_4 \cdot 7H2O$	222 mg
$Na_2MoO_4 \cdot 2H_2O$	1.26 g
$CuSO_4 \cdot 5H_2O$	79 mg
$CoCl_2 \cdot 6H_2O$	40.3 mg

A+ modified 100x

	**1 L**
KCl	60 g
$NaNO_3$	100 g
$CaCl_2 \cdot 2H_2O$	37 g
Na_2EDTA	3 g
KH_2PO_4	5 g

Tris (125 g/L) **pH 8.2 (100x stock)**

$FeCl_3 \cdot 6H_2O$: 30 mM stock in 0.1 M HCl – filter sterile
0.811 g of $FeCl_3 \cdot 6H_2O$ resuspended in 0.1 M HCl to 100 mL

KH_2PO_4: 1 M stock (13.6 g/100 mL MilliQ H_2O) – filter sterile

Vitamin B12: 4 mg/L stock – filter sterile

MAD medium (1 L) preparation protocol (resuspend all ingredients in MilliQ water in the following order):

NaCl	18 g
$MgSO_4 \cdot 7H_2O$	5 g
A^+ modified 100x	10 mL
Tris (125 g/L), pH=8.2	8.3 mL
Modified D7 1000x	1 mL
+ $NaNO_3$	7 g
+ KH_2PO_4 (1 M)	0.83 mL
Autoclave (or sterilize using 0.22 µm filter) and cool down to room temperature to add:	
$FeCl_3 \cdot 6H_2O$ (480 mM)	0.5 mL
Vitamin B_{12}	1 mL

Stock solutions:

Modified D7 metal 1000x

	**1 L**
H_3BO_3	2.86 g
$MnCl_2 \cdot 4H_2O$	1.81 g
$ZnSO_4 \cdot 7H2O$	222 mg
$Na_2MoO_4 \cdot 2H_2O$	1.26 g
$CuSO_4 \cdot 5H_2O$	79 mg
$CoCl_2 \cdot 6H_2O$	40.3 mg

A+ modified 100x

	**1 L**
KCl	60 g
$NaNO_3$	100 g
$CaCl_2 \cdot 2H_2O$	37 g
Na_2EDTA	3 g
KH_2PO_4	5 g

Tris (125 g/L) **pH 8.2 (100x stock)**

$FeCl_3 \cdot 6H_2O$: 480 mM stock in 0.1 M HCl – filter sterile
12.97 g of $FeCl_3 \cdot 6H_2O$ resuspended in 0.1 M HCl to 100 mL

KH_2PO_4: 1 M stock (13.6 g/100 mL MilliQ H_2O) – filter sterile

Vitamin B12: 4 mg/L stock – filter sterile

MAD2 medium (1 L) preparation protocol (resuspend all ingredients in MilliQ water in the following order):

NaCl	18 g
$MgSO_4 \cdot 7H_2O$	5 g
A^+ modified 100x	10 mL
Tris (125 g/L), pH=8.2	8.3 mL
Modified D7 1000x	1 mL
+ $NaNO_3$	15 g
+ KH_2PO_4 (1 M)	2.03 mL
Autoclave (or sterilize using 0.22 µm filter) and cool down to room temperature to add:	
$FeCl_3 \cdot 6H_2O$ (480 mM)	1 mL
Vitamin B_{12}	3 mL

Stock solutions:

<u>Modified D7 metal 1000x</u>

	**1 L**
H_3BO_3	2.86 g
$MnCl_2 \cdot 4H_2O$	1.81 g
$ZnSO_4 \cdot 7H2O$	222 mg
$Na_2MoO_4 \cdot 2H_2O$	1.26 g
$CuSO_4 \cdot 5H_2O$	79 mg
$CoCl_2 \cdot 6H_2O$	40.3 mg

<u>A+ modified 100x</u>

	**1 L**
KCl	60 g
$NaNO_3$	100 g
$CaCl_2 \cdot 2H_2O$	37 g
Na_2EDTA	3 g
KH_2PO_4	5 g

Tris (125 g/L) **pH 8.2 (100x stock)**

$FeCl_3 \cdot 6H_2O$: 480 mM stock in 0.1 M HCl – filter sterile
12.97 g of $FeCl_3 \cdot 6H_2O$ resuspended in 0.1 M HCl to 100 mL

KH_2PO_4: 1 M stock (13.6 g/100 mL MilliQ H_2O) – filter sterile

Vitamin B12: 4 mg/L stock – filter sterile

References:

1. Clark, R. L. *et al.* Light-optimized growth of cyanobacterial cultures: Growth phases and productivity of biomass and secreted molecules in light-limited batch growth. *Metab. Eng.* **47**, 230–242 (2018).
