# Supplementary material for: Newly discovered Synechococcus sp. PCC 11901 is a robust cyanobacterial strain for high biomass production
Source: Commun Biol. 2020 May 7;3:215. doi: 10.1038/s42003-020-0910-8 (PMC7205611; doi:10.1038/s42003-020-0910-8)
Supplement: Supplementary file 2 — Description of Additional Supplementary Items [file 42003_2020_910_MOESM2_ESM.pdf]

Description of Additional Supplementary Files

File Name: **Supplementary Data 1**

Description: Two-tailed t-test analysis and one-way analysis of variation (ANOVA) analysis of the raw datasets.
