# Supplementary material for: Newly discovered Synechococcus sp. PCC 11901 is a robust cyanobacterial strain for high biomass production
Source: Commun Biol. 2020 May 7;3:215. doi: 10.1038/s42003-020-0910-8 (PMC7205611; doi:10.1038/s42003-020-0910-8)
Supplement: Supplementary file 5 — Reporting Summary [file 42003_2020_910_MOESM5_ESM.pdf]

nature research

Corresponding author(s): Artur Włodarczyk, Peter J. Nixon

Last updated by author(s): Mar 2, 2020

Reporting Summary

Nature Research wishes to improve the reproducibility of the work that we publish. This form provides structure for consistency and transparency in reporting. For further information on Nature Research policies, see Authors & Referees and the Editorial Policy Checklist.

Statistics

For all statistical analyses, confirm that the following items are present in the figure legend, table legend, main text, or Methods section.

n/a	Confirmed	
☐	☒	The exact sample size (*n*) for each experimental group/condition, given as a discrete number and unit of measurement
☐	☒	A statement on whether measurements were taken from distinct samples or whether the same sample was measured repeatedly
☒	☐	The statistical test(s) used AND whether they are one- or two-sided<br>*Only common tests should be described solely by name; describe more complex techniques in the Methods section.*
☒	☐	A description of all covariates tested
☒	☐	A description of any assumptions or corrections, such as tests of normality and adjustment for multiple comparisons
☐	☒	A full description of the statistical parameters including central tendency (e.g. means) or other basic estimates (e.g. regression coefficient) AND variation (e.g. standard deviation) or associated estimates of uncertainty (e.g. confidence intervals)
☐	☒	For null hypothesis testing, the test statistic (e.g. *F*, *t*, *r*) with confidence intervals, effect sizes, degrees of freedom and *P* value noted<br>*Give P values as exact values whenever suitable.*
☒	☐	For Bayesian analysis, information on the choice of priors and Markov chain Monte Carlo settings
☒	☐	For hierarchical and complex designs, identification of the appropriate level for tests and full reporting of outcomes
☒	☐	Estimates of effect sizes (e.g. Cohen's *d*, Pearson's *r*), indicating how they were calculated

Our web collection on statistics for biologists contains articles on many of the points above.

Software and code

Policy information about availability of computer code

Data collection	The PacBio reads were assembled using HGAP3 assembler, and then polished with Quiver within the SMRT Analysis v2.3.0 protocol. Polished assembly contigs were then circularized and re-oriented with Circlator 1.1.4. Fluorescence microscopy images images captured using Zen 2.6 lite (Zeiss). Hidex plate reader software version 0.5.35.0. Spectrophotometer software version: Cary WinUV 3.00(182).
Data analysis	MiSeq Illumina paired reads were aligned to the PacBio assembled sequences using the Geneious Prime® 2019.1.3 software (Biomatters Ltd.). Average nucleotide identity (ANI) analysis was performed using OrthoANIu tool (https://www.ezbiocloud.net/tools/ani). Circular diagram of the PCC 11901 genome sequence combined with the BLAST scores of four related cyanobacterial strains compared to PCC 11901 genome was created using CGView Server BETA (http://cgview.ca). Fluorescence microscopy images processed using Zen 2.6 lite (Zeiss). Statistical analysis was done using Excel data analysis internal plugin.

For manuscripts utilizing custom algorithms or software that are central to the research but not yet described in published literature, software must be made available to editors/reviewers. We strongly encourage code deposition in a community repository (e.g. GitHub). See the Nature Research guidelines for submitting code & software for further information.

Data

Policy information about availability of data

All manuscripts must include a data availability statement. This statement should provide the following information, where applicable:

- Accession codes, unique identifiers, or web links for publicly available datasets
- A list of figures that have associated raw data
- A description of any restrictions on data availability

The data that support the findings of this study are available from the corresponding authors upon request. Synechococcus sp. PCC 11901 was deposited in the Pasteur Culture Collection of Cyanobacteria (Paris, France) and is available on request. Complete genome sequence was deposited in GenBank under accession number CP040360.1. Plasmids used for cyanobacterial transformation were deposited in Addgene: pSW036 (ID: 140034), pSW039 (ID: 140035), pSZT025 (ID: 140033), pSW068 (ID: 140036) and pSW071 (ID: 140037). Raw data including all plasmid maps and growth and media optimization data were deposited in fighsare:

https://doi.org/10.6084/m9.figshare.11917893.v1

Field-specific reporting

Please select the one below that is the best fit for your research. If you are not sure, read the appropriate sections before making your selection.

☒ Life sciences ☐ Behavioural & social sciences ☐ Ecological, evolutionary & environmental sciences

For a reference copy of the document with all sections, see nature.com/documents/nr-reporting-summary-flat.pdf

Life sciences study design

All studies must disclose on these points even when the disclosure is negative.

Sample size	Not applicable. Standard deviation calculated based on three biological repeats (and depending on experiment, technical replicates). Only for one FFA producing strain 2 biological duplicates (and 2 technical replicates) were used, due to difficulty in obtaining more positive clones. Statistical analysis was done using ANOVA and two-tailed t-test.
Data exclusions	No data was excluded.
Replication	Experiments were performed using three biological repeats (n=2 for 11901 ΔfadD::tesA strain for one experiment due to difficulty in obtaining more positive clones). For FFA production experiments 2 technical replicates of each biological replicate were measured. Unless otherwise specified, a total of n=3 biological replicates were used to calculate average and standard deviation. Due to low solubility of fatty acids in aqueous buffers, 2 technical replicates were measured for each biological replicate, to minimize the error rate and ensure good data reproducibility throughout the experiment. Replication of all results was successful.
Randomization	Randomization not relevant to this study.
Blinding	Binding not relevant to this study.

Reporting for specific materials, systems and methods

We require information from authors about some types of materials, experimental systems and methods used in many studies. Here, indicate whether each material, system or method listed is relevant to your study. If you are not sure if a list item applies to your research, read the appropriate section before selecting a response.

Materials & experimental systems

n/a	Involved in the study	
☒	☐	Antibodies
☒	☐	Eukaryotic cell lines
☒	☐	Palaeontology
☒	☐	Animals and other organisms
☒	☐	Human research participants
☒	☐	Clinical data

Methods

n/a	Involved in the study	
☒	☐	ChIP-seq
☒	☐	Flow cytometry
☒	☐	MRI-based neuroimaging
